# Effects of Biological Nitrogen Fixation and Nitrogen Deposition on Soil Microbial Communities in Karst Grassland Ecosystems

**DOI:** 10.3390/microorganisms12122429

**Published:** 2024-11-26

**Authors:** Xin Liu, Rong Yang, Jie Zhao, Dan Xiao, Xunyang He, Wei Zhang, Kelin Wang, Hongsong Chen

**Affiliations:** 1 School of Advanced Agricultural Sciences, Weifang University, Weifang 261061, China; xinliu@wfu.edu.cn; 2 Key Laboratory of Agro-Ecological Processes in Subtropical Region, Institute of Subtropical Agriculture, Chinese Academy of Sciences, Changsha 410125, China; yangrong15@mails.ucas.ac.cn (R.Y.); jzhao@isa.ac.cn (J.Z.); danxiao@isa.ac.cn (D.X.); hbhpjhn@isa.ac.cn (X.H.); hbchs@isa.ac.cn (H.C.); 3 Huanjiang Observation and Research Station for Karst Ecosystems, Huanjiang 547100, China; 4 College of Life Sciences, Guangxi Normal University, Guilin 541004, China

**Keywords:** legume plants, nitrogen deposition, soil microbial phospholipid fatty acid, soil C:N ratio, karst area

## Abstract

Diverse exogenous nitrogen (N) sources have a considerable impact on microbial community structure in terrestrial ecosystems. Legume plants and N deposition can relieve N limitations and increase net primary productivity. However, the differences in their effects on soil microbial communities remain unclear. Here, the responses of the soil microbial community to a legume-planting system and simulated N deposition were examined in karst grasslands in Southwest China over five years by analyzing soil microbial phospholipid fatty acids (PLFAs). The experiment included three treatments—legume plant introduction (N_L_, *Indigofera atropurpurea*), N deposition (N_D_, NH_4_NO_3_:10 g N m^−2^ yr^−1^), and a control with no treatment. The effects of N_L_ and N_D_ on soil microbial community composition differed significantly. N_D_ significantly reduced the biomass of bacteria, actinobacteria, and arbuscular mycorrhizal fungi. N_L_ insignificantly increased the biomass of all microbial groups. However, the total amounts of PLFAs and fungal biomass were significantly higher in N_L_ than in N_D_. The effect of legume plant introduction on soil microbial community composition was more powerful than that of N_D_. Overall, the introduction of legume plants is beneficial in terms of increasing the biomass of the soil microbial community and stabilizing the soil microbial community structure in karst grassland ecosystems.

## 1. Introduction

Soil microbes play a crucial role in ecosystem processes by driving the Earth’s biogeochemical cycles [1,2]. This includes soil organic matter (SOM) decomposition, maintenance of plant diversity, and atmospheric concentrations of greenhouse gases [2,3,4,5]. Shifts in the composition and structure of the soil microbial community, as well as the richness of functional genes specific to recalcitrant carbon (C) decomposition in response to nitrogen (N) availability, affect the nutrient cycles [6,7]. Recent meta-analyses have indicated that N addition affects microbial taxa in topsoil in different ways and alters their relative abundances by altering soil pH and N availability [8,9,10]. The amounts of biological N fixation (BNF) and atmospheric N deposition (ND) are estimated to be twice as large as N input throughout natural ecosystems [11,12]. Therefore, the responses of these microbial groups to external N sources such as BNF and atmospheric ND may have a considerable impact on global C and N cycling.

Approximately 80% of the annual BNF worldwide is fixed by legumes [13]. When conditions are conducive to the growth of legumes, such as when grasslands release enough resource space after being disturbed, the symbiotic N fixation rate of leguminous plants is as high as 50 kg N ha^−1^ yr^−1^ [14]. Legume plants are the most important N sources for terrestrial ecosystems and play an important role in the study of BNF [15,16,17]. Legumes positively affect soil N content and availability [18,19,20], as well as soil C accumulation, compared with non-N-fixing species [21]. Soil microbes can be affected by soil C and N availability and plant species richness [22,23,24], and the introduction of legumes is expected to influence soil microbial communities. In a field experiment in subtropical China, Huang et al. [25] found that legumes affected soil microbial communities in the rhizosphere after the establishment of planting systems. Legume plants enhance the structure and complexity of the soil food web, acting as food sources for soil microbes in the forms of leaf litter, dead roots, and root exudates, and may exert bottom-up control over soil microbial communities [26,27,28]. Soil microbes use plant litter and SOM as their sources of C and N, respectively, and the roots of legumes provide extracellular enzymes that help soil microbes break down SOM during plant litter decomposition [29,30]. The introduction of legume plants can influence the soil microbial community structure because fungi and bacteria use organic matter with different C:N ratios, while fungi are the main decomposers of plant residues [31].

The majority of emitted anthropogenic Nr (NH_3_ and NO_y_) components can enter terrestrial and marine ecosystems via atmospheric ND [32,33]. Atmospheric ND is increasing annually worldwide and is expected to rise to 600 Tg N yr^−1^ by 2100 [34,35]. ND can increase plant productivity by enhancing N availability [36,37]. However, excessive ND can induce soil acidification [38,39]; decrease plant diversity [40]; and alter plant biomass C, N, and phosphorus cycles [41,42]. Various effects of ND on soil microbial communities have been reported, including negative [43,44], positive [45], and neutral effects [46]. Long-term N addition decreased soil microbial diversity and changed community composition [44]. Increased N reduces fungal biomass via changes in plant-specific exudates and alterations in nutrient competition between plants and rhizosphere microbes [47]. However, a low-level addition of nitrogen had a short-term positive effect on microbial biomass but no long-term effect [45]. These conflicting results may be due to differences in soil N [48,49], SOM [43,50,51], and pH [48,52,53,54]. N addition altered microbial communities by influencing soil pH and nitrate N (NO^3−^-N) in [8]. Microbial biomass C decreases following N fertilization because of decreased soil pH, resulting in greater osmotic potential and increased solubility of Al, which is toxic to soil microbes [54]. However, Southwest China’s extremely fragile karst ecosystem is one of the largest exposed carbonate rock areas worldwide, at more than 0.54 million km^2^ [55]. The soil of the karst natural grassland ecosystem has high calcium and magnesium contents and a strong acid–base neutralization ability. Within a certain range, pH changes slightly with the addition of soil nitrogen. Karst grassland ecosystems are N-limited [56] and rich in legume plants [57]. The level of total atmospheric ND is high (approximately 3 g N m^−2^ yr^−1^ in this area) [58]. Therefore, the effects of legume plant introduction and ND on the soil microbial communities in karst grassland ecosystems remain unclear. However, relatively few studies have compared their effects on soil physicochemical properties and the soil microbial community.

In this study, we aimed to examine the effects of BNF and ND on soil microbial communities, as indicated by phospholipid fatty acids (PLFAs), in karst grassland ecosystems in Southwest China. The prevailing consensus among researchers is that PLFA analysis cannot be used to identify individual microbial species but can provide an overall fingerprint of the microbial communities found in soils [59]. In addition, since PLFAs are rapidly degraded upon cell death, they can be considered representatives of a viable soil microbial community [59,60]. The sensitivity of soil microorganisms to N enrichment differs among various grassland types [61]. Moreover, the form of applied N has a more significant influence on the soil microbial community than the quantity of N added [61]. Soil microorganisms exhibit distinct responses to different N sources, such as organic nitrogen, ammonium nitrogen, and nitrate nitrogen [8,61]. Based on the results of previous studies, we hypothesized that (1) legume plant introduction would increase the bacterial and fungal biomass and change the soil microbial community composition in karst grassland ecosystems and that (2) ND would suppress the soil microbial community biomass in karst grassland ecosystems.

## 2. Materials and Methods

### 2.1. Study Site and Experimental Design

The study was conducted at the Huanjiang Observation and Research Station for Karst Ecosystems of the Chinese Academy of Sciences (107°51′–108°43′ E, 24°44′–25°33′ N), which is in Huanjiang County, Guangxi Province, Southwest China. These sites experience a typical subtropical monsoon climate, with mean annual temperature and precipitation of 19 °C and 1389 mm, respectively, mainly from May to September. The region in the depression area is a gentle valley surrounded by hills. The brown calcareous soil developed from a dolostone base [62,63]. According to the Chinese Soil Taxonomy system, this is a primitive soil [64]. The soil pH was approximately 7.38, the total atmospheric ND was approximately 37 kg N ha^−1^ yr^−1^ [58], and the NHx-to-NOy deposition ratio was close to 1 [65]. Therefore, this experiment simulated ND by applying an ammonium nitrate (NH_4_NO_3_) solution.

The experimental site was a grassland developed from an abandoned maize–soybean field in 1982. The dominant species were *Apluda mutica*, *Microstegium vagans*, and *Imperata cylindrica*. The experimental area consisted of nine plots (5 × 4 m) in June 2014. All the plots were separated by 15 cm wide concrete walls. The walls were placed 50 cm below the ground to block underground plant roots and the migration of soil moisture and 20 cm above the land surface to prevent surface runoff. The plots were laid out in a randomized block design with three replicate blocks. Each replicate block included three treatments, that is, no nitrogen deposition simulation, no leguminous plant introduction as a control (CK), atmospheric N deposition simulation (N_D_: 10 g N m^−2^ yr^−1^), and a leguminous plant introduction system (N_L_). Since June 2015, 16.6 g of N as NH_4_NO_3_ was dissolved in 1.5 L of tap water and applied monthly to each N_D_ treatment plot near the soil surface using a backpack sprayer. Due to the area’s high rainfall, no water was sprayed onto the CK and N_L_ treatment plots. The leguminous plant used in the present study was *Indigofera atropurpurea*, a native N-fixing shrub in the area. *I. atropurpurea* has the ability to fix atmospheric N through a symbiotic relationship with rhizobium bacteria [57]. We collected seeds of *I. atropurpurea* from the wild and cultivated seedlings. In November 2014, seedlings with the same rhizome and height were transplanted (1 × 1 m) into the N_L_ treatment plots (Table 1). N fixation by *I. atropurpurea* was approximately 16.3 g N m^−2^ yr^−1^ in the N_L_ treatment plots.

### 2.2. Sampling and Physicochemical Analyses

The soil was sampled in July 2014 before treatment application and in July of 2015, 2016, 2017, and 2018. Soil cores (5 cm in diameter) were collected at 0–10 cm depths from five random locations within each plot. Five cores from each plot were combined to form a composite. The litter above each sampling area was removed before the cores were collected. Samples were transported to the laboratory in insulated boxes and passed through a 2 mm sieve to remove stones and roots. Samples for microbial analysis were stored at −20 °C, while the remaining samples were stored under cool conditions during transport to the laboratory. Longer-term storage was at −80 °C for the microbial samples, while all others were stored at +4 °C until processing.

The plants were sampled in October of 2014, 2015, 2016, 2017, and 2018. Two 1 × 1 m subplots were randomly selected and installed to monitor the plant biomass in each 20 m^2^ plot. In October, the ground plant tissues in each subplot were cut, and their fresh weight was measured. Then, a 20 g fresh sample was baked in an oven at 80 °C for 24 h to a constant weight. The plot’s dry weight was measured and converted to plant biomass (kg m^−2^).

Soil pH was measured in deionized water using a glass electrode. Soil organic carbon (SOC, g kg^−1^ dried soil) was measured using the Walkley–Black method, and total N (TN, g kg^−1^ dried soil) was measured after micro-Kjeldahl digestion using a flow injection autoanalyzer (FIA, FIAlab Instruments, Inc., Seattle, WA, USA) [66].

### 2.3. Phospholipid Fatty Acid Analysis

Samples for microbial analysis were stored at −80 °C until they were freeze-dried. Soil microbial community composition and quantity (total biomass) were determined using the phospholipid fatty acid (PLFA) technique based on the Bligh and Dyer solvent extraction procedure. The detailed procedure followed that of Quideau et al. [59] and utilized a surrogate standard of 19:0 (1,2-dinonadecanoyl-sn-glycero-3-phosphocholine, Avanti Polar Lipids Inc., Alabaster, AL, USA) and an instrument standard of 10:0Me (methyl decanoate, Aldrich, St. Louis, MO, USA). Briefly, PLFAs were extracted from 0.5 g of freeze-dried forest-floor sample using the Bligh and Dyer solvent extraction method with a citrate buffer, isolated on SPE columns, converted to methyl esters, and identified and quantified on an Agilent 6890 Series capillary gas chromatograph (GC; Agilent Technologies, Santa Clara, CA, USA) fitted with a 25 m Ultra 2 column (Crosslinked 5% PhMeSilicone) using Sherlock Microbial Identification System Version 4.5 software (MIDI, Inc., Newark, NJ, USA).

Based on previous studies, we selected a suite of fatty acids to represent specific microbial groups [8,59]. The PLFAs used as bacterial biomass were i14:0, 15:0, i15:0, a15:0, i16:0, 16:1ω7c, 17:0, a17:0, i17:0, cy17:0, 18:1ω7c, and cy19:0 (Quideau et al., 2016) [59]. 18:2ω6, 9c, and 18:1ω9c represented fungal PLFAs (Hu et al., 2014) [67,68]. 10 Me 16:0, 10 Me 17:0, and 10 Me 18:0 represented actinomycete PLFAs (Huang et al., 2014) [25,63]. 16:1ω5c indicated arbuscular mycorrhizal fungi (AMF) (Quideau et al., 2016) [59]. Other PLFAs, such as 14:0, 16:0, 17:1w8c, 16:1 2OH, and 16:1ω9c, were also used to analyze the composition of microbial communities. While we used these indices to express biomarker diversity and abundance within treatments, we recognize that differences in PLFA chemical structure may not directly relate to genotypic differences in the microbial community (e.g., greater PLFA chemical diversity may not be linked to greater diversity in microbial species). Instead, our approach considers changes in PLFA chemical structure as an indicator of the microbial community’s broader phenotypic response to changing exogenous N sources.

### 2.4. Statistical Analyses

Data were tested for normality and homogeneity before analysis and were naturally log-transformed when necessary. Repeated analysis of variance (ANOVA) was used to determine the treatment effects of CK, N_D_, and N_L_ on plant biomass, soil chemical properties, and soil microbial communities for each sampling event. Pearson’s correlation analysis was used to identify the relationships between soil microbial communities, plant biomass, and the soil C:N ratio. Repeated-measures ANOVA and Pearson’s correlation analyses were performed using SPSS (version 16.0; SPSS Inc., Chicago, IL, USA). Statistical significance was determined at *p* < 0.05 with the LSD test. The principal response curve (PRC) method was used to determine the temporal trends of soil microbial community composition for each treatment using CANOCO 4.5 (Ithaca). The results are presented as a diagram showing the first principal component of the variance explained by treatment on the *y* axis and the sampling periods on the *x* axis. The control treatment was treated as the zero baseline (horizontal line). The treatment effect is represented by the deviation of each fluctuating line (N_D_, N_L_) from the zero baseline over time. The treatments and sampling times were converted to nominal (0, 1) environmental variables [69]. Two non-parametric multivariate statistical tests of dissimilarity (Adonis and multi-response permutation procedures (MRPPs)) were performed to test the dissimilarity of soil microbial communities between ND and biological nitrogen fixation using the “vegan” package in R statistical software (version 4.3.3) [70].

## 3. Results

### 3.1. Plant Biomass and Soil Chemical Properties

**Figure 1:**
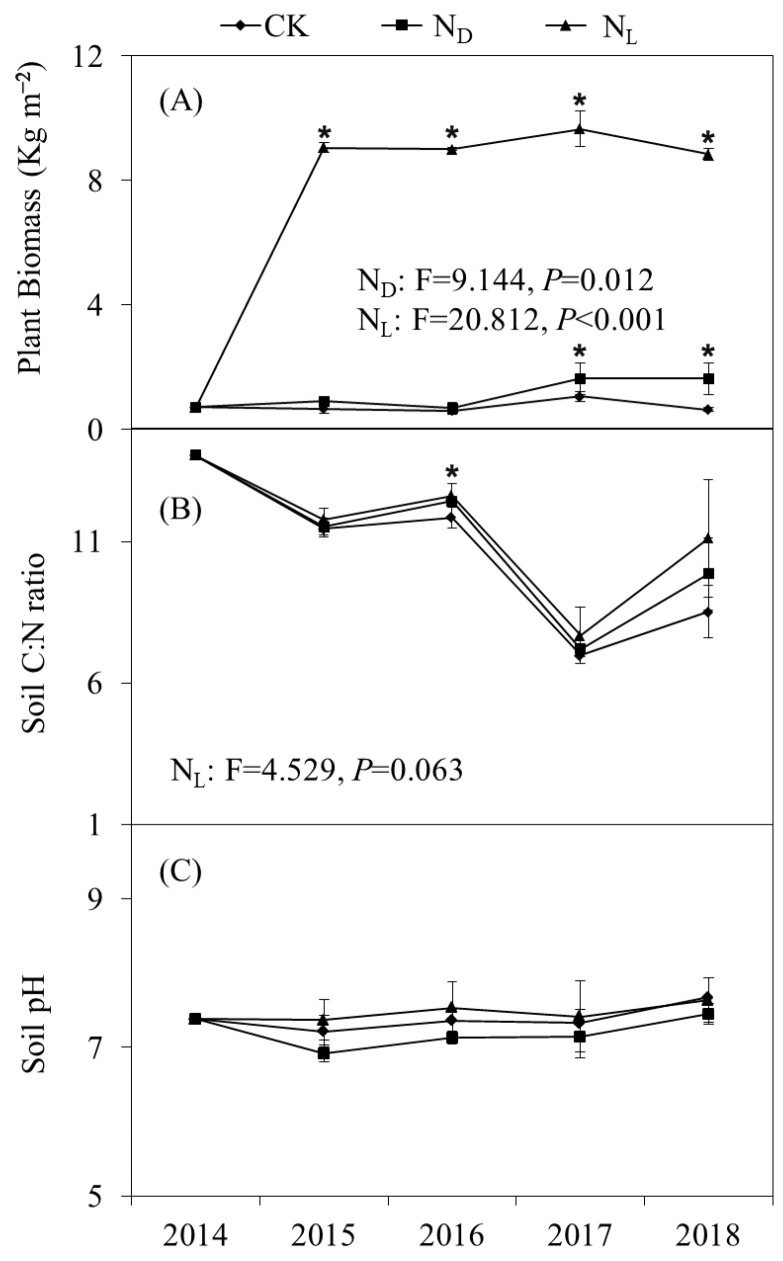
Repeated-measures ANOVA with changes in (**A**) plant biomass; (**B**) soil C:N ratio, i.e., the ratio of soil organic carbon to total nitrogen; and (**C**) soil pH under control (CK), nitrogen deposition (N_D_), and legume plant introduction (N_L_) treatments in each sampling event (2014, 2015, 2016, 2017, and 2018). Bars indicate standard errors of means, and * indicates significance *p* < 0.05.

Repeated-measures ANOVA showed that plant biomass was significantly increased by legume plant introduction and ND and was significantly higher under the treatment with legume plants than under the ND treatment throughout the experimental period (Figure 1A). The introduction of legume plants significantly increased the soil C:N ratio throughout the study period (Figure 1B). Soil pH decreased insignificantly with ND (Figure 1C).

### 3.2. Soil Microbial Community Abundance and Structure

**Figure 2:**
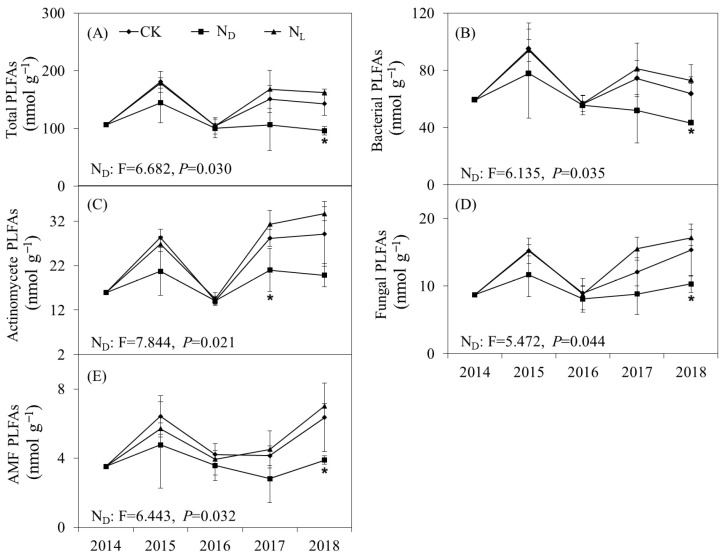
Repeated-measures ANOVA with abundance of (**A**) total phospholipid fatty acids (PLFAs), (**B**) bacterial PLFAs, (**C**) actinomycete PLFAs, (**D**) fungal PLFAs, and (**E**) arbuscular mycorrhizal fungi (AMF) PLFAs, under control (CK), nitrogen deposition (N_D_), and legume plant introduction (N_L_) treatments in each sampling event (2014, 2015, 2016, 2017, and 2018). Bars indicate standard errors of means, and * indicates significance at *p* < 0.05.

**Figure 3:**
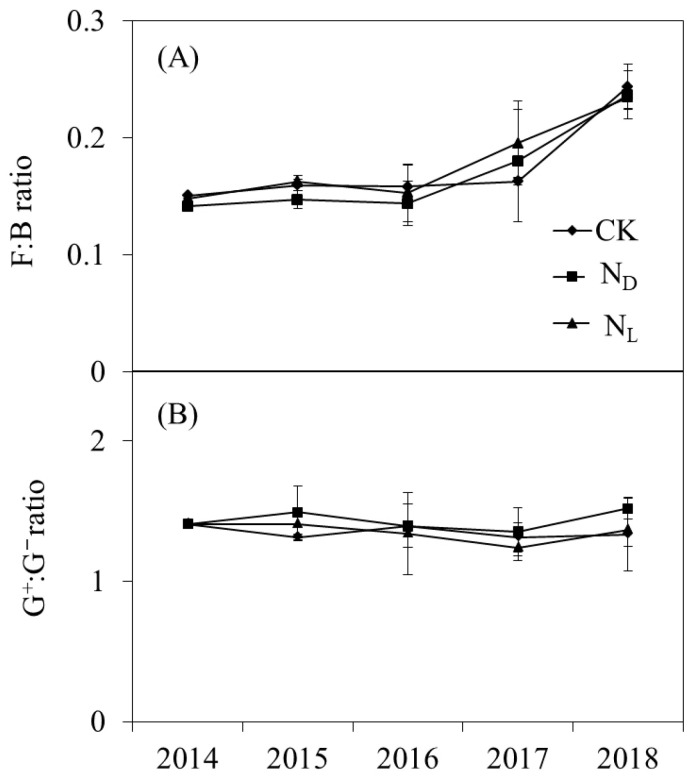
(**A**) Ratio of fungal to bacterial phospholipid fatty acids (PLFAs) (F:B ratio) and (**B**) the ratio of Gram-positive to Gram-negative bacterial PLFAs (G^+^:G^−^ ratio) under control (CK), nitrogen deposition (N_D_), and legume plant introduction (N_L_) treatments in each sampling event (2014, 2015, 2016, 2017, and 2018). Bars indicate standard errors of means.

The abundances of total PLFAs (*p* = 0.030), bacterial PLFAs (*p* = 0.035), actinomycete PLFAs (*p* = 0.021), fungal PLFAs (*p* = 0.044), and AMF PLFAs (*p* = 0.032) were significantly decreased by ND throughout the experimental period (Figure 2). Legume plant introduction had no significant effect on the abundance of the soil microbial community. However, the abundance of the soil microbial community was higher in the legume plant introduction plots than in the ND plots. The ratios of fungal to bacterial PLFAs (F:B ratio) and Gram-positive to Gram-negative bacterial PLFAs (G^+^:G^−^ ratio) were not significantly influenced by ND or legume plant introduction treatments (Figure 3). Legume plant introduction and ND did not significantly affect the soil microbial community structure.

**Figure 4:**
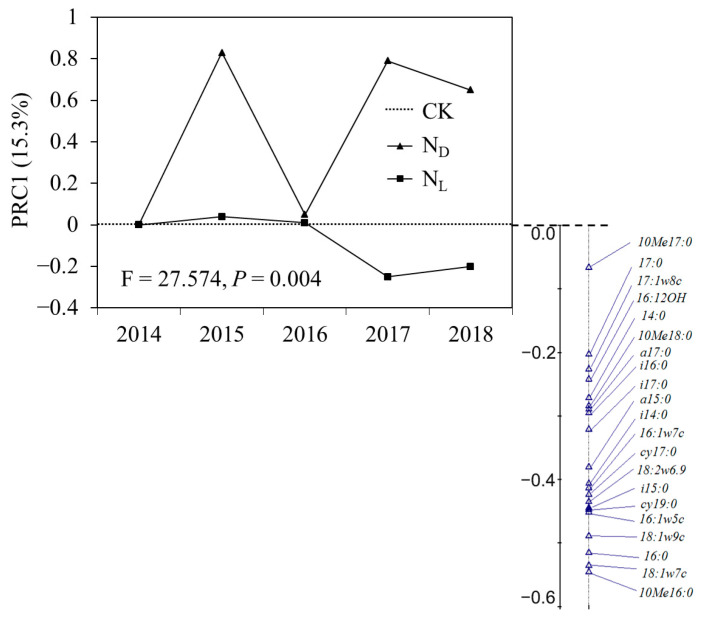
Principal response curves (PRCs) with weights of the density of each soil microbial community under control (CK), nitrogen deposition (N_D_), and legume plant introduction (N_L_) treatments in each sampling event (2014, 2015, 2016, 2017, and 2018). The horizontal axis represents the control treatment.

Adonis and MRPP analyses showed that the effects of ND and legume plant introduction on the soil microbial communities were significantly different (Table 2). PRC analysis showed that the effect of legume plant introduction on the soil microbial community composition was more powerful than the effect of ND (Figure 4). The soil microbial community composition did not respond to the introduction of legume plants in the early years (2014, 2015, and 2016) but showed a positive relationship with legume plant introduction in the fourth (2017) and fifth years (2018). In addition, there was no clear relationship between ND and the soil microbial community composition (Figure 4).

### 3.3. Relationships Between Soil Microbial Variables, Plant Biomass, and Soil C:N Ratio

**Figure 5:**
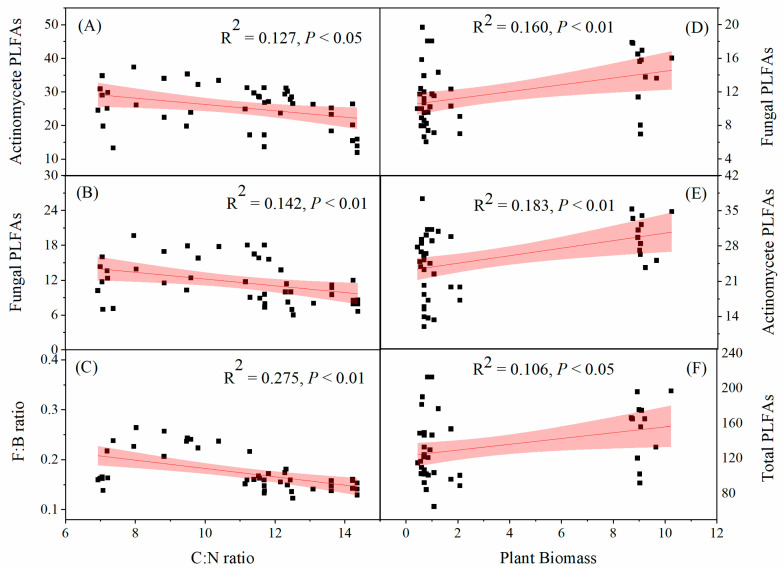
Soil microbial variables as a function of soil C:N ratio and plant biomass. Relationships between (**A**) actinomycete PLFAs, (**B**) fungal PLFAs, (**C**) F:B ratio and C:N ratio, and between (**D**) fungal PLFAs, (**E**) actinomycete PLFAs, (**F**) Total PLFAs and plant biomass. Fitted regressions and their 95% confidence intervals (shaded) and corresponding significance levels (*p*) are presented. F:B ratio: ratio of fungal to bacterial phospholipid fatty acids (PLFAs); C:N: ratio of soil organic carbon to total nitrogen.

The soil C:N ratio exhibited a highly significant negative correlation with fungal PLFAs and the F:B ratio (*p* < 0.01) and a significant negative correlation with actinomycete PLFAs (*p* < 0.05) (Figure 5A–C). Plant biomass exhibited a significant positive correlation with actinomycete PLFAs and fungal PLFAs (*p* < 0.01) and a significant positive correlation with total PLFAs (*p* < 0.05) (Figure 5D–F).

## 4. Discussion

### 4.1. Effects of ND on the Soil Microbial Community

Our study found that bacterial, actinobacterial, and AMF PLFAs were significantly reduced by ND, which is consistent with our hypothesis. Consistent with our study and its findings, most studies have reported that continuous N input negatively affects microbial activity [43]. Tian et al. [71] found that ND significantly negatively affects microbial biomass and community composition in subtropical forests in China. Using high-throughput pyrosequencing, Ramirez et al. [43] found that inorganic N addition consistently altered the microbial community, increasing the relative abundance of Actinobacteria and Firmicutes and decreasing the relative abundance of Acidobacteria and Verrucomicrobia. First, soil acidification caused by ND leads to soil microbial biomass loss [52,72], and soil pH is a significant driver of microbial biomass variation [23,48,53]. Tian et al. [71] found that ND decreased the F:B ratio in subtropical forests, likely due to decreased soil pH. A systematic study across a large pH gradient ranging from 8.3 to 4.5 observed that different microbial species respond differentially to soil pH [73]. The relatively large effect of soil pH on fungal PLFAs compared with the marginal response of bacterial PLFAs to pH results in an F:B ratio pattern similar to that of fungal PLFAs [74]. However, karst regions have a high buffering capacity for soil acidification because the soil is rich in Ca^2+^ and Mg^2+^ [55]. Soil pH was not significantly reduced but was maintained at neutral levels under ND after three years. Compared with NL, soil pH was decreased from 7.37 to 6.92 by ND. ND resulted in a decline in soil pH by 0.3 units compared with the controls. We found that soil pH could not explain the variations in bacterial, actinobacterial, and AMF PLFAs. This indicates that the decrease in these soil microbes in ND may be caused by factors other than soil pH in our study area.

Li et al. [73] found that ND caused significant changes in bacterial diversity and community composition and enriched copiotrophic bacteria. However, it reduced the oligotrophic groups by increasing soil inorganic N content and C availability. The effect of inorganic N addition on soil microbes is related to the C source [43,50,51]. Under ND, the amount of photosynthetic products transferred from the aboveground level to the belowground level decreased. High levels of inorganic N addition decreased fine root production [75] and increased the fraction of recalcitrant compounds such as melanin and lignin [6]. This resulted in less C availability for soil microbes. Williams et al. [51] found that AMF needed to obtain more C from plants under N fertilization. Due to the high energy cost required to obtain a C source, soil microbes may decline with continuous ND [76]. According to the enzyme inhibition hypothesis, N addition could inhibit the enzymes involved in soil recalcitrant C decomposition because soil microbes are mainly C-limited. A decrease in labile C inputs is expected to reduce soil microbial activity and biomass [77,78]. Ramirez et al. [43] found that some soil bacterial groups consistently decreased after one year of N addition in 28 soils in North America. N addition could decrease soil bacterial activities by altering the metabolic capabilities of soil bacterial communities. In our study, the soil C:N ratio exhibited a significant negative correlation with actinomycete PLFAs, fungal PLFAs, and the F:B ratio (Figure 5). Meanwhile, N addition affects fungal growth rates more negatively than bacterial growth rates. Fungal PLFAs decrease more than bacterial PLFAs because of N addition [79]. A strong correlation exists between the fungal growth rate and biomass, but bacterial biomass remains stable when the bacterial growth rate changes significantly [80]. The PRC showed that ND resulted in the biomasses of actinomycetes (10 Me 17:0) and bacteria (15:0) dominating the soil microbial community. ND significantly negatively affected the microbial community structure, owing to the decrease in fungal PLFAs in karst grasslands. Therefore, the significant decreases in bacterial, actinobacterial, and AMF PLFAs under ND may be caused by the soil C:N ratio rather than the soil pH in karst grassland ecosystems. Consequently, against the backdrop of elevated atmospheric ND, enhancing soil carbon availability becomes imperative to counterbalance the detrimental impacts of ND on the microbial communities within karst soils.

### 4.2. Effects of Legumes on the Soil Microbial Community

Legume plant introduction and ND are equivalent to the different exogenous N inputs for soil microbes. The soil microbial community changes with different exogenous N inputs [43,81,82]. Legume plants increase the biomass of soil microbial communities, which is consistent with our hypothesis. Similarly, N-fixers have been reported to exhibit significant positive effects on soil microbial biomass [25,63,83]. For example, legumes increased soil microbial biomass and activity in heavy clay soil near Wageningen [83]. This is mainly because legume plants enhance the structure and complexity of the soil food web by providing main food sources for soil microbes, such as leaf litter, dead roots, and root exudates, which may exert bottom-up control over soil organisms [28,29,30]. Similarly, our research found that legume plants significantly increased plant biomass and the soil C:N ratio (Figure 1) and that plant biomass was significantly positively correlated with total PLFAs (Figure 5). Due to their ability to fix atmospheric N, legume plants have been commonly used to improve net primary production (NPP) and are widely used to improve soil C and N storage [77,84,85]. The soil microbial community is primarily influenced by belowground C and N inputs [25]. Bacteria, fungi, and AMF are attracted to and feed on rhizomes, such as the nutrients, exudates, border cells, and mucilage released by plant roots [86]. Therefore, legume plants can enhance the activities of soil microorganisms. They can alter the soil microenvironment under the canopy, such as the microtopography, soil temperature, humidity, water content, and structure. Dutta and Agrawal [87] found that N-fixing shrubs significantly increased the clay and silt content of tailing soil, reduced the sand content, increased the field water-holding capacity, and reduced soil acidity. The increase in soil structure caused by legume plants can enhance the activity of soil microbes [26,27]. For example, 5-year-old Tectona grandis increased soil microbial C by 92% [27]. The soil respiration rate under the Inga crown cover increased by 15% compared with the control [26]. These results suggest that legume plants play an important role in affecting plant biomass and SOM, with SOC and TN being the two major SOM components, as well as the soil C:N ratio, thereby shaping the composition of the microbial community.

Differences in N_D_ and N_L_ also caused the dominance of fungal PLFAs, that is, 18:1ω7c, in the soil microbial community. This study shows that N_L_ positively affects fungal PLFAs. Legume plants can influence the soil structure, increase soil porosity, reduce soil water content, and make the soil environment conducive to fungal growth [26,27]. In contrast, legume plant tissues have high N contents, and leaf litter, thin roots, and root nodules can reduce the entire litter layer’s C:N ratio and accelerate the litter’s decomposition and turnover. This can provide more C and N sources to soil microbes [41]. He et al. [88] found that the continuous addition of exogenous substrates changed the soil microbial community structure, leading to the alternate growth of bacteria and fungi. Over time, the microbial community became increasingly balanced in meeting C demand. As fungi can effectively decompose inflexible C components, fungal activity is often associated with root systems and large nutrient patches [31,89]. The quantity and structure of fungi are susceptible to plant residues, and the stimulatory effect of plant residues on fungal growth is significantly higher than that on bacteria [31]. In complex soil environments, the response of the microbial community structure to external N sources strongly depends on the availability of C sources and C–N coupling [50]. Compared with ND, legumes improve soil N sources and provide sufficient C sources for soil microbes. This indicates that the introduction of legume plants is beneficial in terms of stabilizing the soil microbial community structure in karst grassland ecosystems.

## 5. Conclusions

ND significantly decreased the abundance of bacteria, Actinobacteria, and AMF PLFAs. The total amounts of PLFAs and bacterial, fungal, actinobacterial, and AMF biomasses were significantly higher in N_L_ than in N_D_. The F:B ratio was not significantly different between N_D_ and N_L_ but showed an increasing trend in N_L_. PRC analysis showed significant differences in the temporal dynamics of the soil microbial community composition within the ND and NL plots. ND negatively affects the soil microbial community by influencing the soil C:N ratio rather than soil pH in karst grassland ecosystems. Legume plants positively affect the soil microbial community through their effects on plant biomass and the C:N ratio of soil. This suggests that it is essential to augment the bioavailability of soil C to mitigate the adverse effects of ND and that the introduction of legume plants into karst grassland ecosystems can increase the biomass of soil microbes and contribute to the maintenance of the stability of the soil microbial community structure. Thus, more attention should be paid to the process of subsurface N transfer by soil microbial networks (such as AMF) in the rhizosphere of legumes to reveal the mechanism whereby legumes promote the stability of the soil microbial community in karst grassland ecosystems.

## Figures and Tables

**Table 1:** Details of treatment plots.

Treatment Types	Treatment Details	Plot Design
Control (CK)	No nitrogen deposition simulation, and no leguminous plant introduction.	Randomized block design with three replicate blocks, each including three treatments (CK, N_D_, and N_L_)
Atmospheric N deposition simulation (N_D_)	NH_4_NO_3_ was dissolved in 1.5 L of tap water and applied monthly to each N_D_ treatment plot near the soil surface.	
Leguminous plant introduction system (N_L_)	Seedlings of *I. atropurpurea* with the same rhizome and height were transplanted (1 × 1 m) into the N_L_ treatment plots.	

**Table 2:** Significance tests using nonparametric multivariate statistical approaches (Adonis and MRPP) to assess the effects of different treatments on soil microbial community.

Comparisons	Adonis			MRPP		
	*R* ^2^	F	*p*	Observed δ	Expected δ	*p*
CK vs. N_D_	0.15	3.871	0.052	0.177	0.187	0.064
CK vs. N_L_	0.016	0.367	0.719	0.146	0.143	0.898
N_D_ vs. N_L_	0.234	6.703	0.005 **	0.167	0.186	0.008 **

CK: control; N_D_: nitrogen deposition; N_L_: legume plant introduction. Differences were considered significant when at least two tests yielded *p* values < 0.05; ** indicates significance at *p* < 0.01.

## Data Availability

All relevant data are included in the manuscript.
